# Supplementary material for: Structure-guided disruption of the pseudopilus tip complex inhibits the Type II secretion in Pseudomonas aeruginosa
Source: PLoS Pathog. 2018 Oct 22;14(10):e1007343. doi: 10.1371/journal.ppat.1007343 (PMC6211770; doi:10.1371/journal.ppat.1007343)
Supplement: S1 Fig — (A) The Type II secretion system consists of multiple protein components that form several subcomplexes. (B) The working model of the secretion of virulence factors in T2SS. The minor pseudopilins form a quaternary pseudopilus tip complex, and the major pseudopilin XcpT polymerizes into piston-like pseudopilus body to secrete substrate (yellow). (PDF) [file ppat.1007343.s001.pdf]

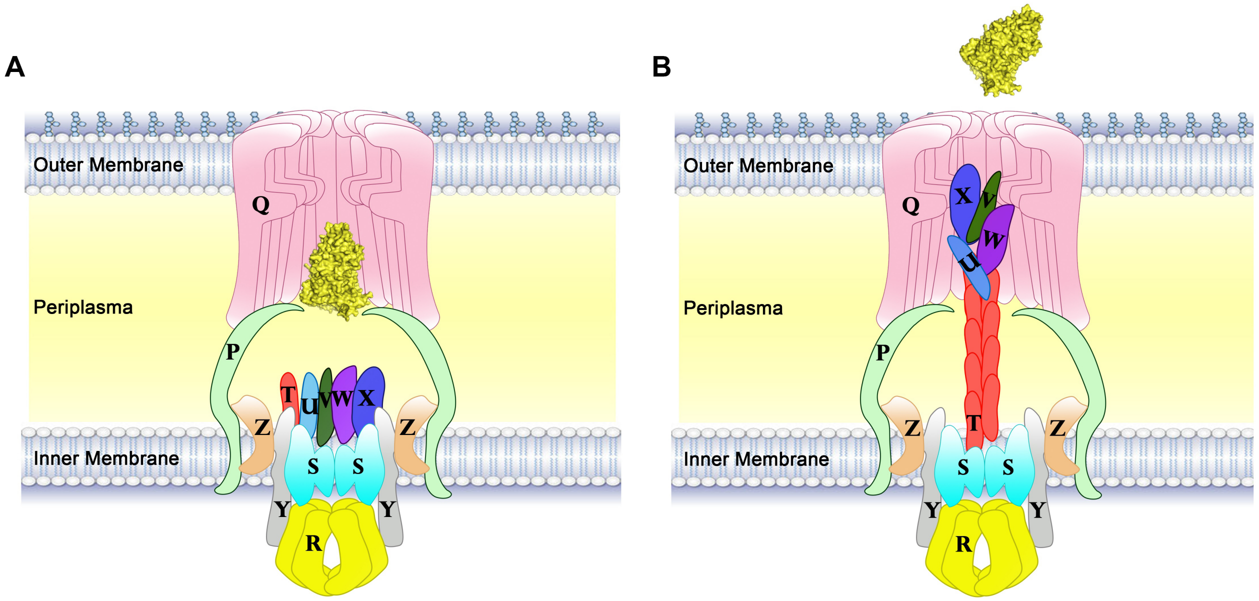

S1 Figure Schematic models of the Type II secretion system. (**A**) The Type II secretion system consists of multiple protein components that form several subcomplexes. (**B**) The working model of the secretion of virulence factors in T2SS. The minor pseudopilins form a quaternary pseudopilus tip complex, and the major pseudopilin XcpT polymerizes into piston-like pseudopilus body to secrete substrate (yellow).
