# Supplementary material for: Structure-guided disruption of the pseudopilus tip complex inhibits the Type II secretion in Pseudomonas aeruginosa
Source: PLoS Pathog. 2018 Oct 22;14(10):e1007343. doi: 10.1371/journal.ppat.1007343 (PMC6211770; doi:10.1371/journal.ppat.1007343)
Supplement: S2 Fig — (A) Comparison of the formation of salt bridges in the XcpVWX (PDB ID:5VTM) and GspIJK complex (PDB ID: 3CI0). The salt bridge is the most important interaction between XcpV(GspI) and XcpX(GspK). In the XcpVWX complex (XcpV in blue and XcpX in yellow), the salt bridge is formed by D51(XcpV) and H49(XcpX) while in the GspIJK complex (GspI in red and GspK in cyan), E45(GspI) and W42(GspK) coordinate to build the salt bridge. (B) Comparison of important interacting residues between the XcpVW complex (XcpV in pink and XcpW in cyan) and the EpsIJ complex (PDB ID: 2RET, EpsI in green, and EpsJ in yellow). Q58(XcpV) and W198(XcpW) form a hydrogen bond to stabilize the interaction between XcpV and XcpW, whereas the hydrophobic interaction between M52(EpsI) and L189(EpsJ) contributes to the interaction. (C) Comparison of important interacting residues between the XcpVW complex (XcpV in pink and XcpW in cyan) and GspIJ in the GspIJK complex (GspI in light green, and XcpW in orange). (D) The residues involved in the interaction between XcpV and -W at the N-termini of the helices. In the XcpVW complex, XcpV and -W also interact through the contacts among residues in the bottom region of the main interface. Hydrogen bonding (D41(XcpV)-Q43(XcpW)) and hydrophobic interactions, i.e. L45(XcpV)-M47(XcpW) and L45(XcpV)—L50(XcpW), are also involved in the binding. (PDF) [file ppat.1007343.s002.pdf]

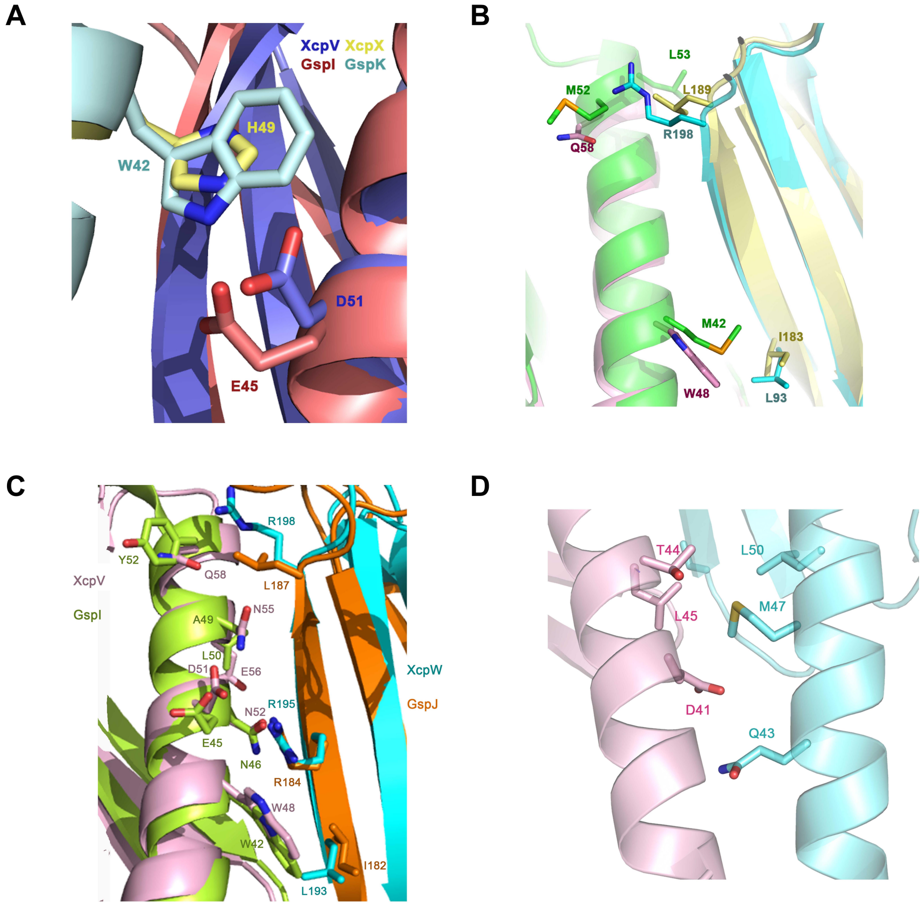


**S2 Figure Protein-protein interactions in minor pseudopilin complexes.** (**A**) Comparison of the formation of salt bridges in the XcpVWX (PDB ID:5VTM) and GspIJK complex (PDB ID: 3CI0)**.** The salt bridge is the most important interaction between XcpV$^{(GspI)}$ and XcpX$^{(GspK)}$. In the XcpVWX complex (XcpV in blue and XcpX in yellow), the salt bridge is formed by D51$^{(XcpV)}$ and H49$^{(XcpX)}$ while in the GspIJK complex (GspI in red and GspK in cyan), E45$^{(GspI)}$ and W42$^{(GspK)}$ coordinate to build the salt bridge. (**B**) Comparison of important interacting residues between the XcpVW complex (XcpV in pink and XcpW in cyan) and the EpsIJ complex (PDB ID: 2RET, EpsI in green, and EpsJ in yellow). Q58$^{(XcpV)}$ and W198$^{(XcpW)}$ form a hydrogen bond to stabilize the interaction between XcpV and XcpW, whereas the hydrophobic interaction between M52$^{(EpsI)}$ and L189$^{(EpsJ)}$ contributes to the interaction. (**C**) Comparison of important interacting residues between the XcpVW complex (XcpV in pink and XcpW in cyan) and GspIJ in the GspIJK complex (GspI in light green, and XcpW in orange). (**D**) The residues involved in the interaction between XcpV and -W at the N-termini of the helices. In the XcpVW complex, XcpV and -W also interact through the contacts among residues in the bottom region of the main interface. Hydrogen bonding (D41$^{(XcpV)}$-Q43$^{(XcpW)}$) and hydrophobic interactions, *i.e.* L45$^{(XcpV)}$-M47$^{(XcpW)}$ and L45$^{(XcpV)}$ - L50$^{(XcpW)}$, are also involved in the binding.
