# Supplementary material for: Structure-guided disruption of the pseudopilus tip complex inhibits the Type II secretion in Pseudomonas aeruginosa
Source: PLoS Pathog. 2018 Oct 22;14(10):e1007343. doi: 10.1371/journal.ppat.1007343 (PMC6211770; doi:10.1371/journal.ppat.1007343)
Supplement: S3 Fig — (A) ITC results show that XcpX’s Kd of calcium binding is 26.04 μM. (B) Phylogenetic trees of GspK(XcpX) proteins in different Gram-negative bacteria illustrate the evolutionary relationship. Left panel: phylogenetic trees of full-length GspKs with the first main clade boxed. Right panel: phylogenetic trees of the sequence of the new calcium-binding site in different GspKs shows that this additional calcium binding site only exists in limited Gram-negative bacteria (highlighted by the dotted box). (C) The alignment of the sequences of canonical calcium binding sites in different species implies that several species do not possess the ability of binding calcium. All the necessary residues for calcium binding are labeled. (PDF) [file ppat.1007343.s003.pdf]

A

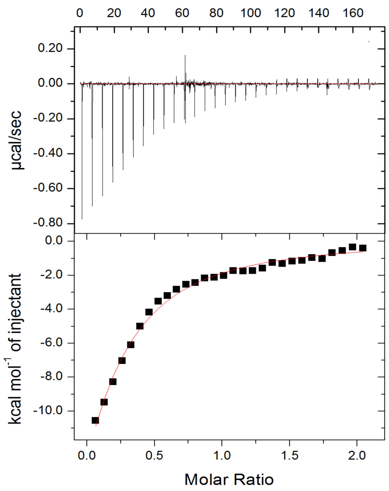

Enthalpy	ΔH= $-5.54\times10^{4}\pm2.38\times10^{4}$ cal/mol
Entropy	ΔS=165 cal/mol/degree
K_a	$3.84\times10^{4}\pm4.93\times10^{3}$ M^{-1}
K_d	26.04µM

B

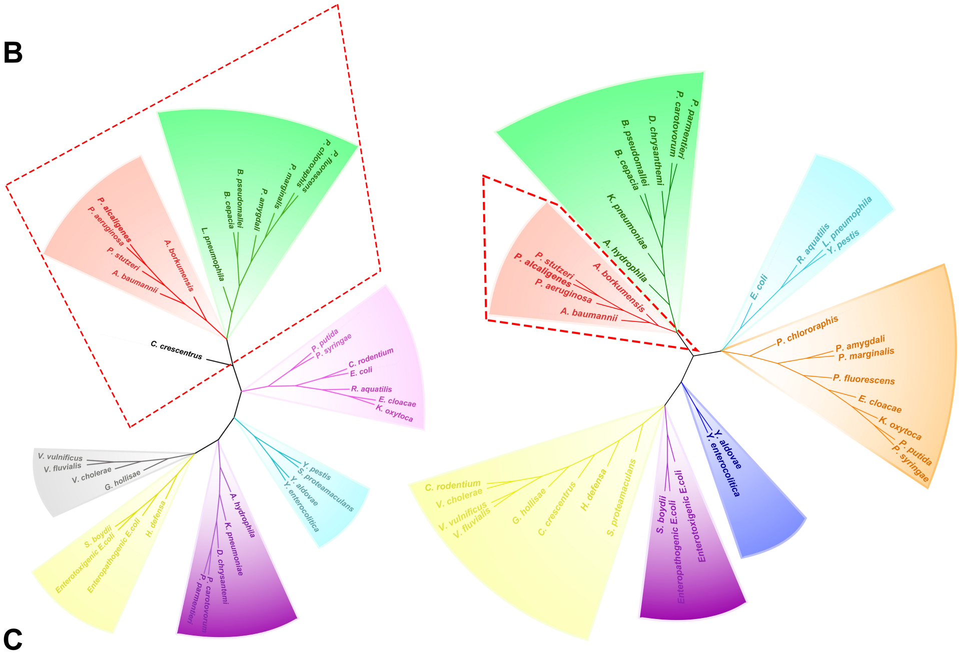

C

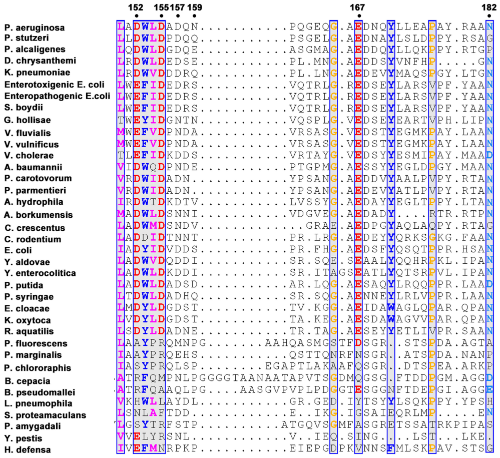

S3 Figure XcpX exhibits calcium-binding activity. (**A**) ITC results show that XcpX's K_d of calcium binding is 26.04 μM. (**B**) Phylogenetic trees of $GspK^{(XcpX)}$ proteins in different Gram-negative bacteria illustrate the evolutionary relationship. Left panel: phylogenetic trees of full-length GspKs with the first main clade boxed. Right panel: phylogenetic trees of the sequence of the new calcium-binding site in different GspKs shows that this additional calcium binding site only exists in limited Gram-negative bacteria (highlighted by the dotted box). (**C**) The alignment of the sequences of canonical calcium binding sites in different species implies that several species do not possess the ability of binding calcium. All the necessary residues for calcium binding are labeled.
