# Supplementary material for: Structure-guided disruption of the pseudopilus tip complex inhibits the Type II secretion in Pseudomonas aeruginosa
Source: PLoS Pathog. 2018 Oct 22;14(10):e1007343. doi: 10.1371/journal.ppat.1007343 (PMC6211770; doi:10.1371/journal.ppat.1007343)
Supplement: S4 Fig — (A) Schematics of the truncations of XcpU. The full-length of XcpU contains 172 residues, in which 29–172 is the soluble domain that is used in our research. Different substructures have been highlighted in different colors to demonstrate the truncation forms of XcpU that were cloned and used for pull-down assay. XcpU structures are modeled based on the structure of GspH (PDB ID: 2KNQ). We truncated the C-terminal β-sheet and N-terminal α-helix to identify the interacting region with XcpW. (B) Results of GST pull-down indicate that the N-terminal α-helix is responsible for the binding of XcpU to XcpW. The clones that have the N-terminal α-helix maintained the ability of binding with XcpW (lane 4–6) even with the loss of the C-terminal β-sheet. On the contrary, when the N-terminal α-helix was truncated, all the clones lost the affinity for XcpW (lane 7–9). (PDF) [file ppat.1007343.s004.pdf]

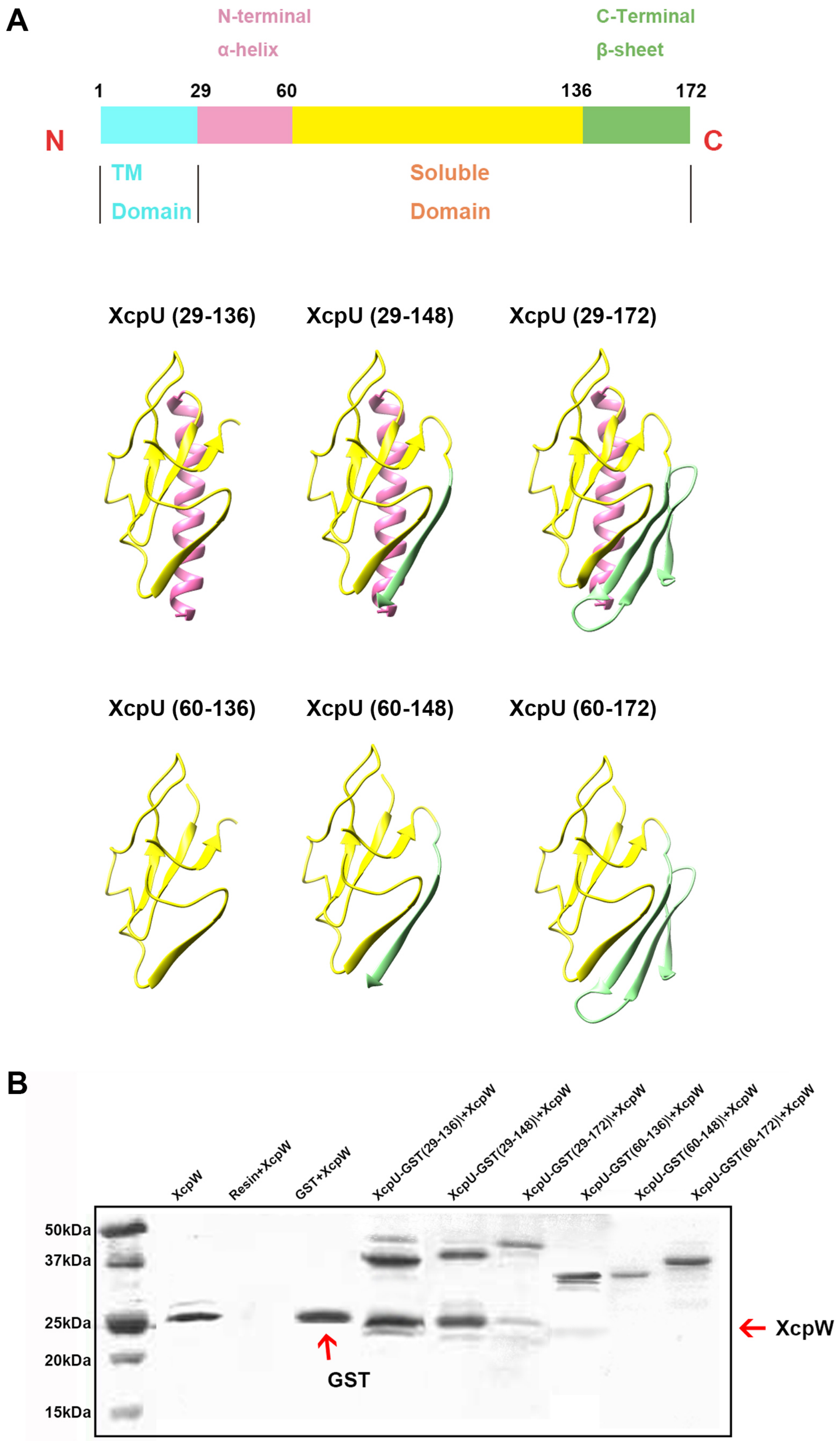
A
N-terminal
α-helix
C-Terminal
β-sheet
1
29
60
136
172
N
C
TM
Domain
Soluble
Domain
XcpU (29-136)
XcpU (29-148)
XcpU (29-172)
XcpU (60-136)
XcpU (60-148)
XcpU (60-172)
B
XcpW
Resin+XcpW
GST+XcpW
XcpU-GST(29-136)+XcpW
XcpU-GST(29-148)+XcpW
XcpU-GST(29-172)+XcpW
XcpU-GST(60-136)+XcpW
XcpU-GST(60-148)+XcpW
XcpU-GST(60-172)+XcpW
50kDa
37kDa
25kDa
20kDa
15kDa
GST
XcpW

S4 GST pull-down assays show that the N-terminal α-helix is crucial for the interaction between XcpU and XcpW. Schematics of the truncations of XcpU. The full-length of XcpU contains 172 residues, in which 29-172 is the soluble domain that is used in our research. Different substructures have been highlighted in different colors to demonstrate the truncation forms of XcpU that were cloned and used for pull-down assay. XcpU structures is modeled based on the structure of GspH (PDB ID: 2KNQ). We truncated the C-terminal β-sheet and N-terminal α-helix to identify the interacting region with XcpW. (**B**) Results of GST pull-down indicate that the N-terminal α-helix is responsible for the binding of XcpU to XcpW. The clones that have the N-terminal α-helix maintained the ability of binding with XcpW (lane 4-6) even with the loss of the C-terminal β-sheet. On the contrary, when the N-terminal α-helix was truncated, all the clones lost the affinity for XcpW (lane 7-9).
