# Supplementary material for: Structure-guided disruption of the pseudopilus tip complex inhibits the Type II secretion in Pseudomonas aeruginosa
Source: PLoS Pathog. 2018 Oct 22;14(10):e1007343. doi: 10.1371/journal.ppat.1007343 (PMC6211770; doi:10.1371/journal.ppat.1007343)
Supplement: S5 Fig — The spatial arrangement of the XcpVWX ternary complex in the envelope is shown. There is extra space for accommodating another molecule, suggesting that the envelope contains all of the four pseudopilins. (PDF) [file ppat.1007343.s005.pdf]

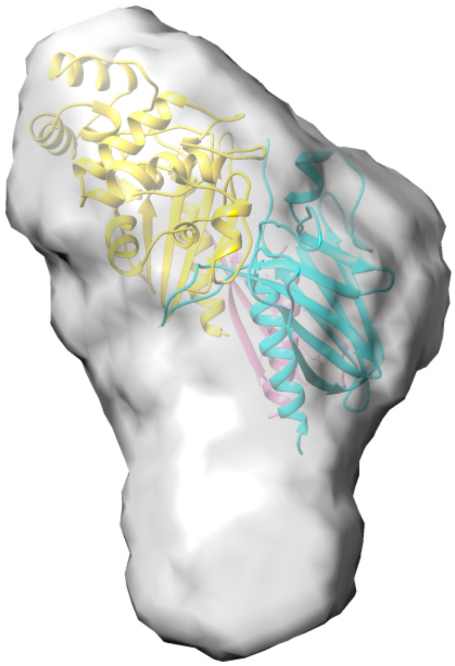

S5 Spatial arrangement of minor pseudopilin molecules based on the SAXS envelope. The spatial arrangement of the XcpVWX ternary complex in the envelope is shown. There is extra space for accommodating another molecule, suggesting that the envelope contains all of the four pseudopilins.
