# Supplementary material for: Structure-guided disruption of the pseudopilus tip complex inhibits the Type II secretion in Pseudomonas aeruginosa
Source: PLoS Pathog. 2018 Oct 22;14(10):e1007343. doi: 10.1371/journal.ppat.1007343 (PMC6211770; doi:10.1371/journal.ppat.1007343)
Supplement: S6 Fig — Plasmids containing XcpV and -W were internalized back to xcpV or -W mutant strains respectively and restored the (A) secretion pattern of T2SS, (B) secretion of lipase, and (C) secretion ring of skim milk clearance, which indicate that T2SS has been recovered in mutant strains. (PDF) [file ppat.1007343.s006.pdf]

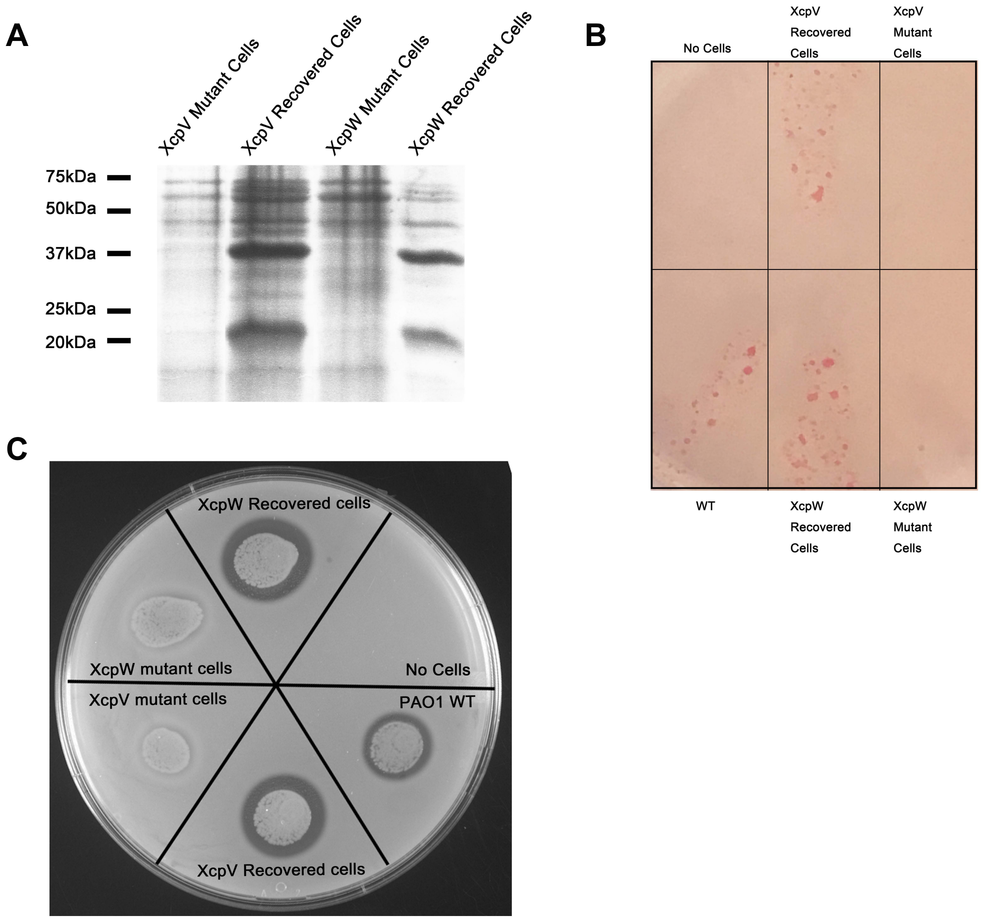

S6 Figure Complementary assays confirm that recovery of XcpV and -W expression in secretion-deficiency strains restored T2SS. Plasmids containing XcpV and -W were internalized back to *xcpV* or -*W* mutant strains respectively and restored the (**A**) secretion pattern of T2SS, (**B**) secretion of lipase, and (**C**) secretion ring of skim milk clearance, which indicate that T2SS has been recovered in mutant strains.
