# Supplementary material for: Structure-guided disruption of the pseudopilus tip complex inhibits the Type II secretion in Pseudomonas aeruginosa
Source: PLoS Pathog. 2018 Oct 22;14(10):e1007343. doi: 10.1371/journal.ppat.1007343 (PMC6211770; doi:10.1371/journal.ppat.1007343)
Supplement: S7 Fig — (A) Correlation factor reveals the most dynamic region in the XcpVW complex. The correlation factor of the XcpVW complex after a 50-ns MD simulation indicates the range of most dynamic residues (highlighted in the red box). During the simulation, all the key residues responsible for the interaction have experienced little change, maintaining stable binding. (B) Cartoon schematic shows that the dynamic region is located at the top of the complex (yellow), which suggests that the region could be targeted by inhibitory molecules. (C) Surface of the interaction interface in the complex. The interacting residues are shown in the interface for one protein component, while another protein is depicted in surface representation, and vice versa. (D) MD simulation (50 ns) of XcpV and -W in the presence of the peptides. Left panel: MD simulation (50 ns) shows the changes of the association between the peptides and pseudopilins. Binding of Peptide 1 to XcpW exhibits the binding free energy of -24.26 kcal/mol, which is weakened and further reduced to -0.92 kcal/mol due to angle change (107.3°). Right panel: during the simulation, the binding free energy changed from -34.26 kcal/mol to -24.13 kcal/mol. The flexibility allows Peptide 2 to influence the conformation of XcpV, which even induces conformational change at the N-terminus. (PDF) [file ppat.1007343.s007.pdf]

A

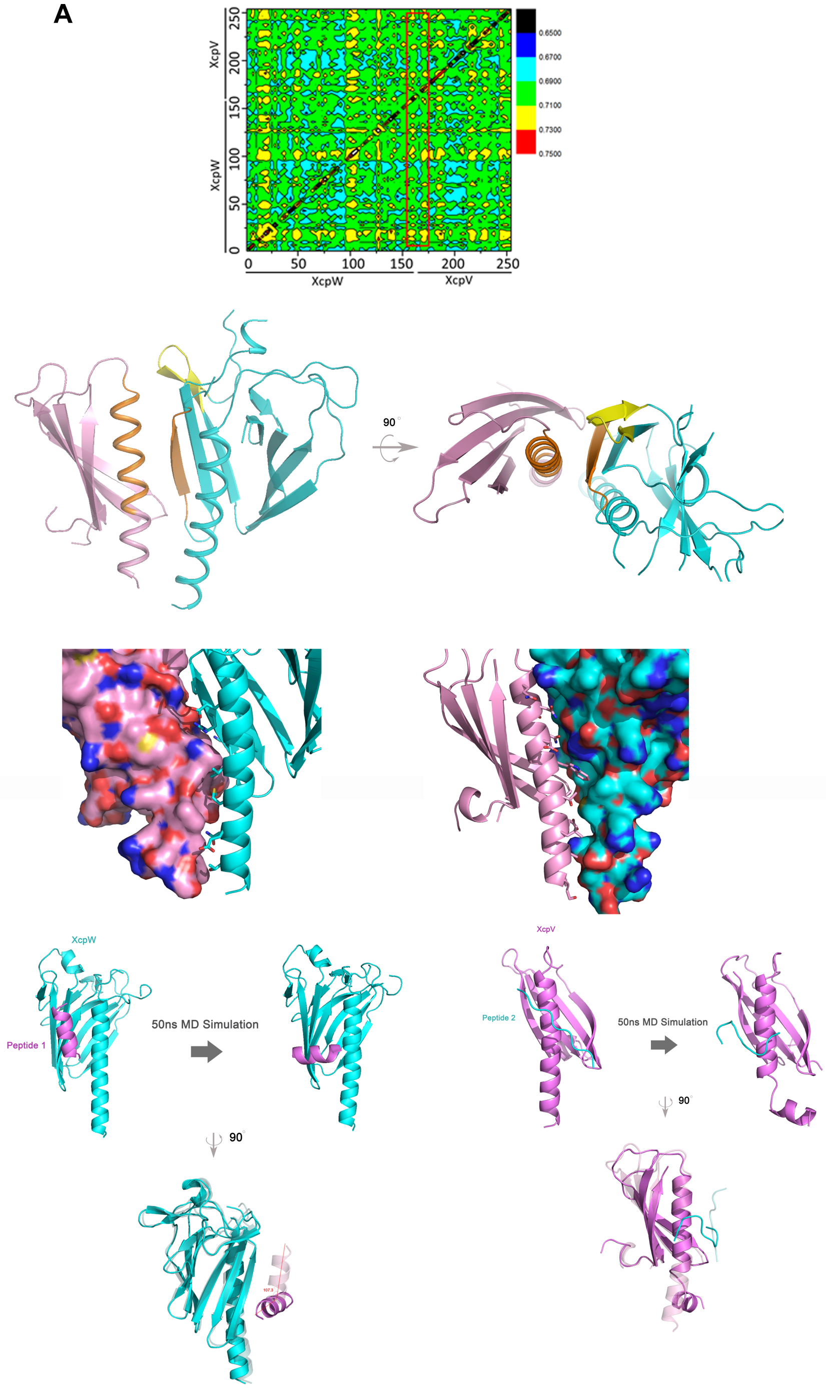

S7 Figure Molecular dynamics simulations of the XcpVW complex and the interaction between the peptides and pseudopilins. (**A**) Correlation factor reveals the most dynamic region in the XcpVW complex. The correlation factor of the XcpVW complex after a 50-ns MD simulation indicates the range of most dynamic residues (highlighted in the red box). During the simulation, all the key residues responsible for the interaction have experienced little change, maintaining stable binding. (**B**) Cartoon schematic shows that the dynamic region

is located at the top of the complex (yellow), which suggests that the region could be targeted by inhibitory molecules. (**C**) Surface of the interaction interface in the complex. The interacting residues are shown in the interface for one protein component, while another protein is depicted in surface representation, and *vice versa*. (**D**) MD simulation (50 ns) of XcpV and -W in the presence of the peptides. Left panel: MD simulation (50 ns) shows the changes of the association between the peptides and pseudopilins. Binding of Peptide 1 to XcpW exhibits the binding free energy of -24.26 kcal/mol, which is weakened and further reduced to -0.92 kcal/mol due to angle change (107.3º). Right panel: during the simulation, the binding free energy changed from -34.26 kcal/mol to -24.13 kcal/mol. The flexibility allows Peptide 2 to influence the conformation of XcpV, which even induces conformational change at the N-terminus.
