# Supplementary material for: Structure-guided disruption of the pseudopilus tip complex inhibits the Type II secretion in Pseudomonas aeruginosa
Source: PLoS Pathog. 2018 Oct 22;14(10):e1007343. doi: 10.1371/journal.ppat.1007343 (PMC6211770; doi:10.1371/journal.ppat.1007343)
Supplement: S8 Fig — (A) Each minor pseudopilin was individually expressed and purified using Ni2+-NTA resin followed by size exclusion chromatography using Superdex 75 column, after removal of the Trx tag. Each pseudopilin produces a single peak corresponding to monomer of the expected molecular weight. (B) Formation of different minor pseudopilin complexes was observed in size exclusion chromatography, and confirmed by SDS-PAGE. (C) The major AUC peak corresponds to the quaternary complex of ~80 kDa, confirming the formation of the XcpUVWX complex. (PDF) [file ppat.1007343.s008.pdf]

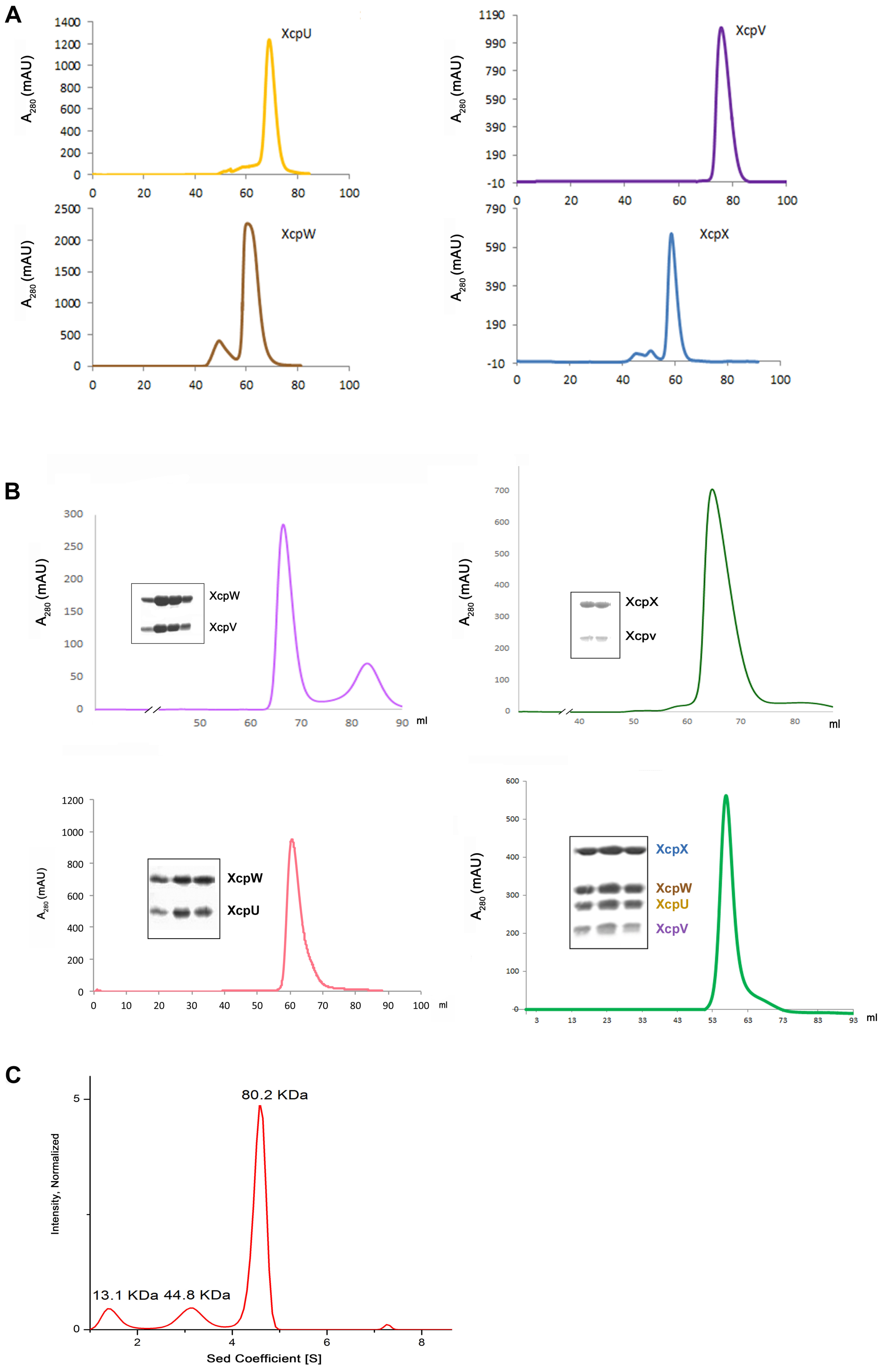

A
XcpU
XcpV
XcpW
XcpX
A280 (mAU)
B
XcpW
XcpV
XcpX
Xcpv
XcpW
XcpU
XcpX
XcpW
XcpU
XcpV
ml
C
80.2 KDa
13.1 KDa 44.8 KDa
Intensity, Normalized
Sed Coefficient [S]

S8 Figure Chromatographic profiles of individual minor pseudopilins and analytical ultracentrifugation (AUC) profile of the XcpUVWX quaternary complex. (**A**) Each minor pseudopilin was individually expressed and purified using Ni^{2+}-NTA resin followed by size exclusion chromatography using Superdex 75 column, after removal of the Trx tag. Each pseudopilin produces a single peak corresponding to monomer of the expected molecular weight. (**B**) Formation of different minor pseudopilin complexes was observed in size exclusion chromatography, and confirmed by SDS-PAGE. (**C**) The major AUC peak corresponds to the quaternary complex of ~80 kDa, confirming the formation of the XcpUVWX complex.
