# Supplementary material for: Structure-guided disruption of the pseudopilus tip complex inhibits the Type II secretion in Pseudomonas aeruginosa
Source: PLoS Pathog. 2018 Oct 22;14(10):e1007343. doi: 10.1371/journal.ppat.1007343 (PMC6211770; doi:10.1371/journal.ppat.1007343)
Supplement: S9 Fig — (PDF) [file ppat.1007343.s009.pdf]

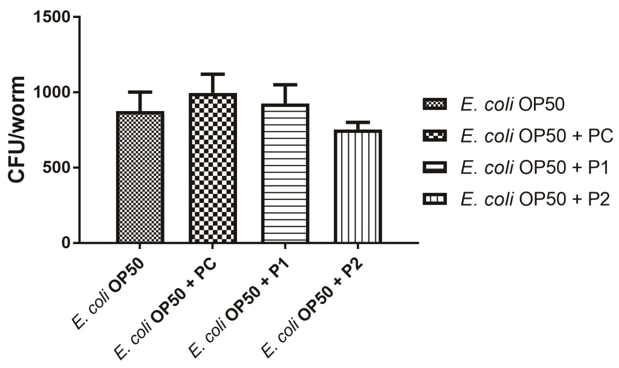

S9 Figure. Structure-based peptides that inhibit the T2SS of *P. aeruginosa* do not affect *E. coli* colonization in *C. elegans*.
