# Supplementary material for: Structure-guided disruption of the pseudopilus tip complex inhibits the Type II secretion in Pseudomonas aeruginosa
Source: PLoS Pathog. 2018 Oct 22;14(10):e1007343. doi: 10.1371/journal.ppat.1007343 (PMC6211770; doi:10.1371/journal.ppat.1007343)
Supplement: S1 Table — (PDF) [file ppat.1007343.s010.pdf]

S1 Table. SAXS data collection and processing statistics

	XcpUVWX
q-range (Å^{-1})	0.0107 – 0.2767
Guinier R_g (Å)	30.02
$p(r)$ R_g (Å)	30.02
$I(0)$	0.023
D_{max} (Å)	96.34
Porod Volume (Å^3)	126,000
Estimated Molecular Weight (kDa)	83.1
Theoretical Molecular Weight (kDa)	80.3
