# Supplementary material for: Structure-guided disruption of the pseudopilus tip complex inhibits the Type II secretion in Pseudomonas aeruginosa
Source: PLoS Pathog. 2018 Oct 22;14(10):e1007343. doi: 10.1371/journal.ppat.1007343 (PMC6211770; doi:10.1371/journal.ppat.1007343)
Supplement: S2 Table — (PDF) [file ppat.1007343.s011.pdf]

S2 Table. Transposon Mutant Strains used in the Experiments

Strain Name	Location	PA ORF	Gene Abbrev.	Position in ORF	Forward Primer Sequence	Reverse Primer Sequence
PW6203	lacZwp09q1D01	PA3097	*xcpX*	738(1002)	AGAACAGGCAACTGCCCTC	CGTTTCAACCTCAACGGACT
PW6204	lacZwp09q3E07	PA3097	*xcpX*	271(1002)	ACGGCATAACTGACGTTTCC	CTCGACAAGGAGCACAACTG
PW6206	phoAwp07q2A07	PA3098	*xcpW*	26(714)	CTCCAGGGTCATCTCCACC	CGAATTGCAACTGGAACAGA
PW6205	lacZwp02q1G09	PA3098	*xcpW*	18(714)	CTCCAGGGTCATCTCCACC	CGAATTGCAACTGGAACAGA
PW6208	phoAwp06q2H02	PA3099	*xcpV*	240(390)	GTCCAGAACCAGCCAGTAGC	GCGAGAAGGAGCAGAAGAAA
PW6207	lacZwp05q3G01	PA3099	*xcpV*	74(390)	TCCAGAACCAGCCAGTAGC	GCGAGAAGGAGCAGAAGAAA
PW6210	phoAbp03q1B01	PA3100	*xcpU*	434(519)	CTGTCGAACATGCGGTAGGT	ATCGAGCTGATGGTGGTGAT
PW6209	lacZbp01q3E10	PA3100	*xcpU*	26(519)	CCAGTTGCAATTCGTTGAGA	GCTGGACAACTTCGCCTATC
