# Supplementary material for: Structure-guided disruption of the pseudopilus tip complex inhibits the Type II secretion in Pseudomonas aeruginosa
Source: PLoS Pathog. 2018 Oct 22;14(10):e1007343. doi: 10.1371/journal.ppat.1007343 (PMC6211770; doi:10.1371/journal.ppat.1007343)
Supplement: S1 Methods — (PDF) [file ppat.1007343.s012.pdf]

Supporting Methods

Isothermal titration calorimetry

XcpX calcium binding measurement using ITC was performed following the protocol as described[1]. The protein solution was loaded in the sample cell, and $CaCl_2$ solution was loaded in the syringe. The concentration of XcpX ranged from 20 μM to 60 μM; it was titrated by $CaCl_2$ with 10-fold in concentration at room temperature. The heat released per injection was measured, and all the data were analyzed using the Microcal Origin ITC software.

GST Pull-down Assay

The truncation forms of XcpU were constructed into pGEX-4T-3 vector for fusion with N-terminal GST tag. Truncations were expressed in Codon Plus competent cells at 18 °C with 0.5 μM of IPTG overnight. The GST tagged truncations of XcpU were purified using standard GST protein purification protocol[2,3]. Purified proteins were concentrated and loaded onto Superdex 75 for further SEC purification. GST pull-down assay was conducted following the reported procedures[4]. The amounts of protein used in the system were 10 ng for XcpU truncations and 12 ng for XcpW for full saturation. The binding of XcpU truncations and XcpW was carried out at 4 °C for 2 h followed by washing with 1 ml of GST buffer with agitation. The resins were washed three times and then taken for SDS-PAGE.

Sequence searching, alignment and phylogenetic tree generation

Sequences of GspK proteins of different Gram-negative bacteria, including pathogenic and non-pathogenic species, were searched and obtained on Uniprot database (http://www.uniprot.org/). Using CLUSTAL Omega[5,6], all the full sequences of GspK were aligned while maintaining gaps and output in the format of clustal with numbers. The scripts of tree file were also obtained which were further applied to drawing phylogenetic tree on iTOL (https://itol.embl.de/)[7]. An unrooted phylogenetic tree that reflects the relationship of GspKs in different species was generated by the iTOL server. The leaves on the same clade were classified into the same group, and different groups were separated in colors.

The aligned sequence was submitted to Espript 3.0 server[8] (http://espript.ibcp.fr/ESPript/ESPript/) to analyze the similarities among different sequences. Referring to the results, the novel calcium binding site and the canonical calcium binding site were identified. The sequences of novel binding site were subjected to further alignment and phylogenetic tree generation.
