# Supplementary material for: Structure-guided disruption of the pseudopilus tip complex inhibits the Type II secretion in Pseudomonas aeruginosa
Source: PLoS Pathog. 2018 Oct 22;14(10):e1007343. doi: 10.1371/journal.ppat.1007343 (PMC6211770; doi:10.1371/journal.ppat.1007343)
Supplement: S1 References — (PDF) [file ppat.1007343.s013.pdf]

References

1 Radhakrishnan, A., Stein, A., Jahn, R. & Fasshauer, D. The Ca2+ affinity of synaptotagmin 1 is markedly increased by a specific interaction of its C2B domain with phosphatidylinositol 4,5-bisphosphate. *The Journal of biological chemistry* **284**, 25749-25760, doi:10.1074/jbc.M109.042499 (2009).

2 Harper, S. & Speicher, D. W. Purification of proteins fused to glutathione S-transferase. *Methods Mol Biol* **681**, 259-280, doi:10.1007/978-1-60761-913-0_14 (2011).

3 Einarson, M. B., Pugacheva, E. N. & Orlinick, J. R. Preparation of GST Fusion Proteins. *CSH Protoc* **2007**, pdb prot4738, doi:10.1101/pdb.prot4738 (2007).

4 Einarson, M. B., Pugacheva, E. N. & Orlinick, J. R. GST Pull-down. *CSH Protoc* **2007**, pdb prot4757, doi:10.1101/pdb.prot4757 (2007).

5 Sievers F, W. A., Dineen DG, Gibson TJ, Karplus K, Li W, Lopez R, McWilliam H, Remmert M, Söding J, Thompson JD, Higgins D. Fast, scalable generation of high-quality protein multiple sequence alignments using Clustal Omega. *Molecular Systems Biology*.

6 Goujon, M. *et al.* A new bioinformatics analysis tools framework at EMBL-EBI. *Nucleic acids research* **38**, W695-699, doi:10.1093/nar/gkq313 (2010).

7 Letunic, I. & Bork, P. Interactive tree of life (iTOL) v3: an online tool for the display and annotation of phylogenetic and other trees. *Nucleic acids research* **44**, W242-245, doi:10.1093/nar/gkw290 (2016).

8 Robert, X. & Gouet, P. Deciphering key features in protein structures with the new ENDscript server. *Nucleic acids research* **42**, W320-324, doi:10.1093/nar/gku316 (2014).
